# Effect of Limestone Particle Size and Potassium Supplementation on Growth Performance, Blood Physiology, and Breast Muscle Myopathy of Male Broiler Chickens

**DOI:** 10.3389/fvets.2020.603284

**Published:** 2020-12-15

**Authors:** Dinabandhu Joardar, Kimberly A. Livingston, Frank W. Edens, Basheer Nusairat, Rasha Qudsieh, Matthew L. Livingston, John Brake

**Affiliations:** ^1^Prestage Department of Poultry Science, NC State University, Raleigh, NC, United States; ^2^Department of Animal Production, College of Agriculture, Jordan University of Science and Technology, Irbid, Jordan

**Keywords:** limestone particle size, potassium, blood physiology, woody breast, white striping, broiler, performance

## Abstract

The experiment investigated the effects of limestone particle size and dietary potassium (K) on live performance, blood physiology, and muscle myopathies in broilers raised to 35 days of age. A total of 384 Ross male broilers were placed in 24 floor pens and fed four diets during the starter (0–16 days of age) and grower (17–33 days of age) periods containing two limestone particle sizes (fine: 0.2 mm and coarse: 0.9 mm), and amended with either 0% basal K (K–) or 0.2% added dietary K (K+) as potassium carbonate to complete the 2 × 2 factorial arrangement. Live performance was measured from 1–33 days of age. Blood physiology, woody breast (WB), and white striping (WS) scores were measured at 35 days of age. The K+ dietary treatment reduced (*P* < 0.05) feed intake and BWG when compared to K– during the starter and grower period. The K+ dietary treatment decreased blood Na (mmol/L), blood glucose (mg/dl), ionized blood Ca (mg/dl), TCO_2_ (mmol/L), blood HCO_3_ (mmol/L), and base excess in extracellular fluid (mmol/L) when compared to K– birds of similar body weight at 35 days of age (*P* ≤ 0.05). Fine limestone diets tended to reduce WB scores (3.0 vs. 2.59) when compared to coarse limestone diets at 35 days of age (*P* = 0.08). This study demonstrated that using 0.2% of K as potassium carbonate did not negatively affect FCR even though FI and BWG were reduced. Furthermore, fine limestone has the potential to reduce WB in breast muscle tissues; however, further research is needed to confirm these outcomes.

## Introduction

Wooden breast (WB) and white striping (WS) are two forms of muscle myopathies that have emerged in the last decade. These myopathies have been associated with fast growing, high-yield broiler chickens (1, 2), and were shown to be potentially associated with acid–base imbalances (3). The multiple ion channels of sodium (Na), potassium (K), chloride (Cl), calcium (Ca), and magnesium (Mg) play a critical role in proper muscle function (4). The intracellular fluid (ICF) major cation K, as well as Na and Cl, the major ions of extracellular fluid (ECF) play important regulatory roles (5) in avian acid–base homeostasis. The relative changes in concentration of Na, K, and Cl in the blood can offset metabolic or respiratory acid–base imbalances through buffering mechanisms in blood gas and kidney (6). The decrease in blood ionized Ca (iCa) causes loss of stability in muscle cell membranes characterized by muscle twitching, and the release of Ca during muscle contraction upregulates glycolysis (7) where glucose status in ICF is maintained via phosphorylation of glucose. The Ca-mediated changes in permeability of muscle membranes are caused by dysregulation of Ca homeostasis and acid–base imbalances and, along with the altered glucose metabolism, are often associated with WB and WS myopathies (8, 9). Hence, blood biochemical parameter measures are optimum indicators to detect the physiological state of acid–base balance, and the blood variables involved in the assessment of physiological disturbances, which are pH, partial pressure of CO_2_ (pCO_2_), and blood HCO_3_, as well as hemoglobin concentration (10, 11). Furthermore, the blood iCa and glucose are indicators of metabolic and physiological status used for assessment of breast muscle myopathies in chickens (3, 12, 13).

The dietary electrolyte balance of the diets (DEB, as a formula of Na + K–Cl expressed as mEq/kg) (14) is usually calculated and balanced during diet formulation to maximize the efficiency of digestive and metabolic processes. Previous studies have demonstrated that when DEB was manipulated by K salts, potassium chloride (KCl) improved growth performance, meat quality, and plasma K status (15, 16), while potassium carbonate (K_2_CO_3_) has demonstrated a close relationship between supplemental K, blood physiology, and breast muscle myopathies, WB and WS (3).

Limestone, a mineral composed of calcium carbonate (CaCO_3_), is the most widely used source of Ca supplementation in the animal feed industry. A wide range of limestone particle sizes and solubilities are used. Recently, the effects of limestone solubility and particle size on growth performance, Ca, phosphorus (P) and amino acids utilization has garnered much interest in broiler nutrition (17, 18). However, there is a lack of information on muscle and blood physiology parameters as affected by limestone particle size and its interaction with K salt supplementation in broiler chickens. Therefore, this study was designed to explore the interaction between dietary limestone particle size and levels of K on live performance, blood physiology, and breast muscle WB and WS scores of broiler chickens.

## Materials and Methods

All practices of animal care and experimental procedures used in this study were reviewed and approved by the North Carolina State University Institutional Animal Care and Use Committee. A total of 384 Ross 708 male broilers chicks were individually tagged and placed in 24 pens (1.2 × 1.8 m; 4.8 m^2^) with a stocking density of 0.135 m^2^/bird. The mean body weight (BW) at placement in each pen was 45 ± 0.95 g. Each pen had two tube feeders and one Plasson water drinker. Pens were bedded with fresh pine shavings (20 cm deep). Floor temperature at chick placement was initially 35.6°C, then reduced to 32°C within 24 h. Air temperature was then reduced gradually each day until 7 days of age where it reached 29°C and was maintained at that temperature until 14 days. Temperature was then reduced to approximately 27°C from 15 to 21 days and 24°C from 16 to 33 days. Artificial light was provided by 18-W fluorescent bulbs. The lighting program during the first 7 days was 23 h of light, which was then reduced to 22 h of light to 14 days, and 20 h of light to 21 days. After 21 days of age, only 15 h of light was used.

### Experimental Diets

Broilers were assigned to one of four corn–soybean meal-based dietary treatments that were fed in the starter (days 0–16) and grower (days 17–33) feeding phases (Table 1) in a 2 × 2 factorial arrangement. The factors were limestone particle sizes of 0.2 mm (fine) and 0.9 mm (coarse), containing either existing basal levels of K (K–) or 0.2% added K (K+) as potassium carbonate (56.5% K concentration; Arm&Hammer Animal Nutrition). Each treatment had six replicate pens and 16 male broilers per pen (blocked by location within the house). Diets were formulated to either meet or exceed the nutrient requirements for poultry (19). Samples of each treatment diet were collected after mixing and post-pelleting, and were analyzed for particle size (20) and proximate analysis for nutrients (moisture, crude protein, crude fat, crude fiber, and ash) as well as K, Na, and, Cl [using inductively coupled plasma-optical emission spectroscopy (ICP-OES)] following AOAC official methods. The particle size distributions of the two sources of limestone were also determined using ASAE S319.3 method (19). Experimental diets were supplemented with dietary Monensin USP−90 mg/kg feed (Coban 90, Elanco US, Inc., Greenfield, IN, USA) for coccidiosis control.

**Table 1:** Composition of experimental starter and grower diets.

	**Starter^a^**				**Grower^b^**			
	**Fine limestone^c^**		**Coarse limestone^d^**		**Fine limestone^c^**		**Coarse limestone^d^**	
**Ingredients (%)**	**K–**	**K+**	**K–**	**K+**	**K–**	**K+**	**K–**	**K+**
	(%)								
Corn	58.50	58.50	58.50	58.50	65.00	65.00	65.00	65.00
Soybean meal (48% CP)	31.50	31.50	31.50	31.50	26.00	26.00	26.00	26.00
Poultry by-product meal	5.00	5.00	5.00	5.00	4.00	4.00	4.00	4.00
Poultry fat	2.00	2.00	2.00	2.00	2.50	2.50	2.50	2.50
Limestone	0.50	0.50	0.50	0.50	0.36	0.36	0.36	0.36
Defluorinated phosphate	1.05	1.05	1.05	1.05	0.77	0.77	0.77	0.77
Salt	0.30	0.30	0.30	0.30	0.30	0.30	0.30	0.30
Potassium carbonate^e^	0.00	0.20	0.00	0.20	0.00	0.20	0.00	0.20
Sand	0.27	0.07	0.27	0.07	0.24	0.04	0.24	0.04
Choline chloride (60%)	0.20	0.20	0.20	0.20	0.10	0.10	0.10	0.10
Vitamin premix^f^	0.05	0.05	0.05	0.05	0.05	0.05	0.05	0.05
Mineral premix^g^	0.20	0.20	0.20	0.20	0.20	0.20	0.20	0.20
Selenium premix^h^	0.05	0.05	0.05	0.05	0.05	0.05	0.05	0.05
DL-Methionine	0.20	0.20	0.20	0.20	0.20	0.20	0.20	0.20
L-Lysine	0.11	0.11	0.11	0.11	0.11	0.11	0.11	0.11
L-Threonine	0.07	0.07	0.07	0.07	0.07	0.07	0.07	0.07
Coccidiostat^i^	0.05	0.05	0.05	0.05	0.05	0.05	0.05	0.05
Total	100	100	100	100	100	100	100	100
**Calculated nutrient content**								
Metabolizable energy (kcal/g)	2.99	2.99	2.99	2.99	3.09	3.09	3.09	3.09
Crude protein	23.38	23.38	23.38	23.38	20.54	20.54	20.54	20.54
Calcium	0.90	0.90	0.90	0.90	0.71	0.71	0.71	0.71
Available phosphorus	0.45	0.45	0.45	0.45	0.36	0.36	0.36	0.36
Digestible lysine	1.16	1.16	1.16	1.16	1.01	1.01	1.01	1.01
Digestible methionine	0.53	0.53	0.53	0.53	0.50	0.50	0.50	0.50
Digestible methionine+cysteine	0.80	0.80	0.80	0.80	0.75	0.75	0.75	0.75
Digestible threonine	0.79	0.79	0.79	0.79	0.70	0.70	0.70	0.70
Sodium	0.25	0.25	0.25	0.25	0.23	0.23	0.23	0.23
Chloride	0.28	0.28	0.28	0.28	0.26	0.26	0.26	0.26
Potassium	0.92	1.03	0.92	1.03	0.80	0.92	0.80	0.92
DEB^j^ (mEq/kg)	266	291	266	291	231	262	231	262
**Analyzed nutrient content**								
Crude protein	22.58	20.92	23.22	20.50	20.68	21.39	21.26	22.03
Ether extract	4.25	4.35	4.16	4.31	5.10	5.44	5.38	5.43
Crude fiber	3.00	2.70	2.60	2.60	2.40	2.30	2.40	2.50
Ash	4.88	4.90	5.21	5.08	4.84	4.42	4.78	4.06
Total digestible nutrients	86.19	87.40	86.34	86.32	89.54	91.31	90.51	90.76
Sodium	0.16	0.15	0.17	0.18	0.16	0.13	0.18	0.14
Chloride	0.23	0.29	0.26	0.22	0.25	0.22	0.23	0.22
Potassium	0.75	0.87	0.79	0.90	0.67	0.76	0.65	0.70
Analyzed DEB^k^ (mEq/kg)	197	206	203	246	170	189	180	178

a *Starter diet was fed at 16 days of age*.

b *Grower diet was fed from approximately 16 to 35 days of age*.

c *Fine limestone particle size, 0.2 mm*.

d *Coarse limestone particle size, 0.9 mm*.

e *Potassium carbonate (as 56.5% K): (K–) basal levels of K; (K+) 0.20% K as potassium carbonate (starter and grower)*.

f *Vitamin premix supplied the following per kg of diet: 13,200 IU vitamin A, 4,000 IU vitamin D3, 33 IU vitamin E, 0.02 mg vitamin B12, 0.13 mg biotin, 2 mg menadione (K3), 2 mg thiamine, 6.6 mg riboflavin, 11 mg d-pantothenic acid, 4 mg vitamin B6, 55 mg niacin, and 1.1 mg folic acid*.

g *Mineral premix supplied the following per kg of diet: manganese, 120 mg; zinc, 120 mg; iron, 80 mg; copper, 10 mg; iodine, 2.5 mg; and cobalt, 1 mg*.

h *Selenium premix provided 0.2 mg Se (as Na2SeO3) per kg of diet*.

i *Coccidiostat supplied monensin sodium at 90 mg/kg of food*.

j *Dietary electrolyte balance calculated using matrix values of sodium, chloride, and potassium*.

k *Dietary electrolyte balance calculated using analyzed values sodium, chloride, and potassium*.

### Live Performance and Blood Physiology

Pen body weight (BW) and feed consumed (FI) were recorded at placement, 16 days, and 33 days for calculation of average BW, BWG, and FI per bird for each replicate pen. Mortality was recorded, necropsied to determine the cause of death, and weighed daily to account for the BW of all deceased birds, total BWG, and total FI in mortality-adjusted feed conversion ratio (FCR) calculations for each dietary feeding phase. At 35 days of age, average BW per pen was determined. Additionally, for venous blood analysis, a total of 64 birds were selected (within 200 g of the pen average) from four replicate pens per each dietary treatment (four birds per pen per treatment).

Blood was drawn and analyzed individually from these birds. Approximately 2 ml of blood was drawn from the left ulnaris vein and analyzed using the i-Stat® handheld blood analyzer fitted with a CG8+ cartridge (Abbott Point of Care Inc., Princeton, NJ, USA), which measured packed cell volume (PCV), hemoglobin (Hgb), ionized calcium (iCa), glucose (Glu), Na, K, pH, partial pressure of carbon dioxide (pCO_2_), bicarbonate (HCO_3_), total carbon dioxide (TCO_2_), base excess in the extracellular fluid (BEecf), partial pressure of oxygen (pO_2_), and oxygen saturation of hemoglobin (sO_2_) (21). A systematic approach was utilized to catch bird, draw and analyze blood, and return the birds back to their respective pens to assure uniform collection of blood and handling of the birds.

### Carcass Measurements

A WB and WS scoring system developed for the *Pectoralis* major breast muscle was used to determine myopathies This subjective one to four-point ordinal scale of measurement has been previously described by Livingston et al. (22). This WB scoring system comprised a hand palpation method where a score of 1 indicated normal or no signs of WB. A score of 2 indicated some firming or hardening of the breast with over 50% of non-affected tissue being pliable. A WB score of 3 indicated that more than 50% of the breast was hard and resisted palpation but with some pliability still present. A WB of 4 indicated no presence of pliability and over 90% of the breast was hard to the touch. The WS scoring system was similar with a score of 1 for normal breast tissue or no signs of striping. A WS score of 2 indicated a mild amount of visible striations, a WS score of 3 indicated a moderate amount of striping, and a WS score of 4 indicated severe striations across the ventral portion of the boneless skinless breast fillet. At 35 days of age, all birds in the 24 pens with 16 birds/pen were euthanized by cervical dislocation and were allowed to proceed through rigor mortis (23). WB and WS scoring were conducted 6 h postmortem.

### Statistical Analysis

All data collected in this study were analyzed as a randomized block design (RBD), with a 2 × 2 arrangement of treatments. The two factors were limestone particle size (fine vs. coarse) and with or without added K (K– or K+) observed in each of the two complete blocks. Accordingly, the following linear mixed effects model was used for data analysis, *Y*_ijk_ = μ + α_i_ + β_j_ + (αβ)_ij_ + *B*_k_ + ε_ijk_ where i, j, and k are indices for limestone particle size, K, and block, respectively. Here *B*_k_ denotes a random block effect. The data were analyzed using the Fit Model Platform of JMP 13.2 software (24). Growth performance data were analyzed using pen as the experimental unit, while individual birds were considered the experimental unit for blood chemistry, WB and WS scores. Significance level was set at *P* ≤ 0.05, and a tendency for significance was set at *P* ≤ 0.10. Standard least squares were separated using Tukey's test for multiple comparisons. To further discern the venous blood chemistry variables, bivariate regression analysis was performed with Fit Y by X Platform of JMP 13.2. Significance was determined at *P* ≤ 0.05. Further analysis of the data was performed using WB and WS scores as independent variables with the same dependent blood variables with the Fit Model Platform of JMP 13.2. Significance was determined when *P* ≤ 0.05.

## Results

### Growth Performance

The effects of dietary treatments on FI, BWG, and FCR are shown in Table 2. No significant differences were observed in the live performance between limestone dietary treatments. Adding K+ to starter and grower feed decreased the feed intake and BWG when compared to those chicks fed with the K- diets (*P* < 0.05). However, no differences were observed on cumulative FCR to 33 days of age in either of the dietary treatments.

**Table 2:** Effect of limestone particle size and dietary potassium (K) on feed intake (FI), body weight (BW), BW gain (BWG), and feed conversion ratio (FCR) from 1–16 days to 1–33 days of age.

**Limestone^1^**	**Potassium^2^**	**Age period (days)**					
		**1–16**			**1–33**		
		**FI**	**BWG**	**FCR**	**FI**	**BWG**	**FCR**
		**g/bird**		**(g:g)**	**g/bird**		**(g:g)**
Fine		749	560	1.33	3,101	2,194	1.41
Coarse		750	551	1.36	3,146	2,208	1.43
	K–	763^A^	567^A^	1.34	3,172^A^	2,235^a^	1.42
	K+	736^B^	544^B^	1.35	3,074^B^	2,167^b^	1.42
Fine	K–	760	571	1.33	3,126	2,205	1.42
Fine	K+	737	550	1.34	3,074	2,182	1.41
Coarse	K–	766	563	1.36	3,219	2,264	1.42
Coarse	K+	736	538	1.36	3,073	2,151	1.43
	SEM^3^	9.6	7.7	0.017	34.7	30.2	0.011
		(Probability > F)					
Limestone		0.85	0.22	0.12	0.20	0.64	0.28
Potassium		<0.01	<0.01	0.64	0.01	0.03	0.91
Limestone × Potassium		0.67	0.83	0.91	0.67	0.15	0.50

a,b *Means within a column lacking a common superscript differ significantly (P < 0.05)*.

A,B *Means within a column lacking a common superscript differ significantly (P < 0.01)*.

1 *Limestone particle size (starter and grower); Fine (0.2 mm) and coarse (0.9 mm)*.

2 *Potassium carbonate (starter and grower); (K–) basal levels of K; (K+) 0.20% added K*.

3 *SEM, Standard error of mean for n = 6 pens for each interaction effect*.

### Blood Physiology

**Figure 1:**
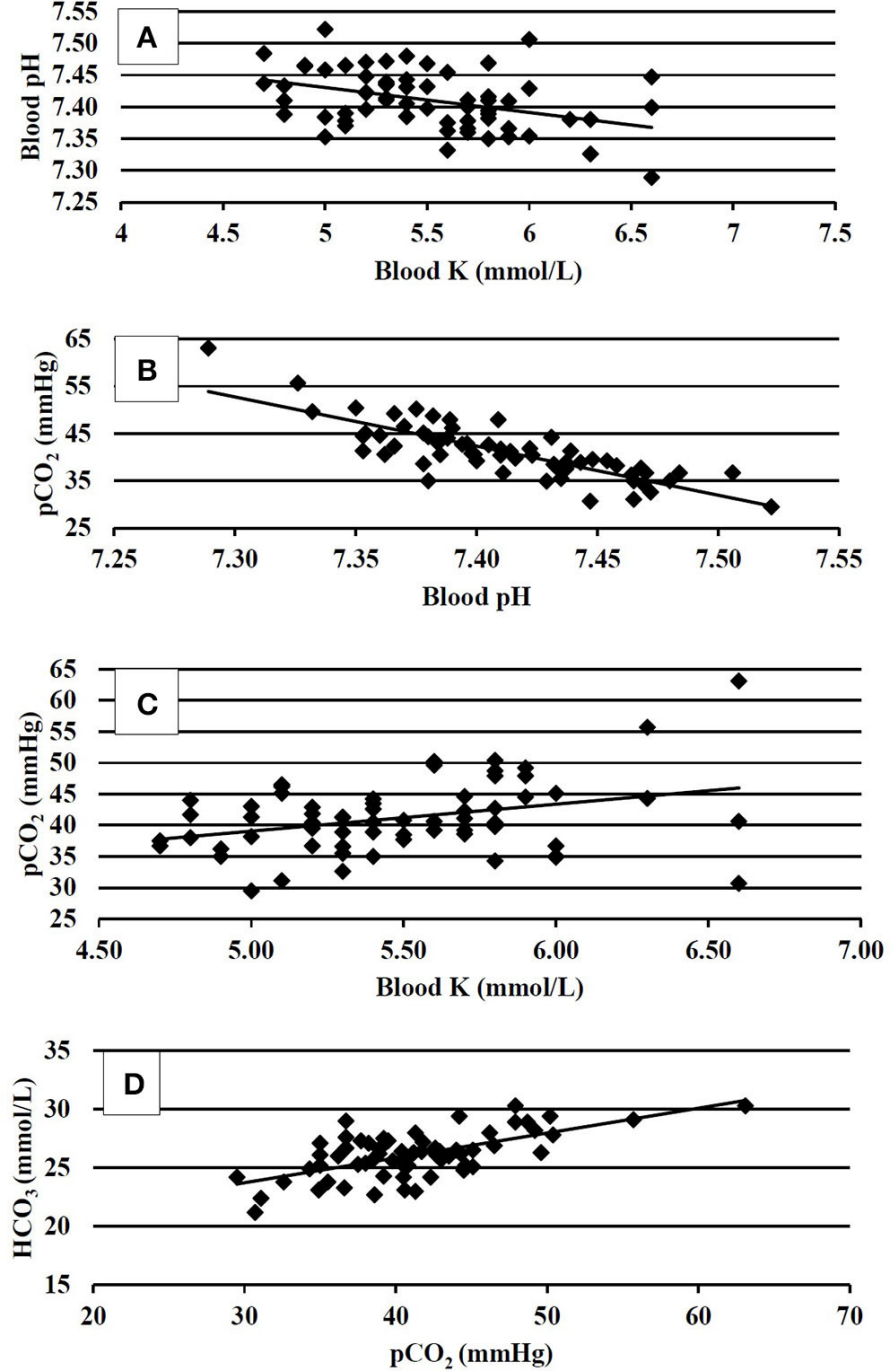
**(A)** Correlation between venous blood pH and venous blood K at 35 days of age, *P* < 0.01; *R*^2^ = 0.15. **(B)** Correlation between venous blood pCO_2_ and venous blood pH at 35 days of age, *P* < 0.01, *R*^2^ = 0.69. **(C)** Correlation between venous blood pCO_2_ and venous blood K at 35 days of age, *P* < 0.01; *R*^2^ = 0.13. **(D)** Correlation between venous blood HCO_3_ and venous blood pCO_2_ at 35 days of age, *P* < 0.01; *R*^2^ = 0.42.

Blood physiology results at 35 days of age are shown in Table 3. Broilers that had been fed with K+ diets had significantly decreased (*P* < 0.05) blood Na (mmol/L), blood glucose (mg/dl), blood iCa (mg/dl), TCO_2_ tension (mmol/L), blood HCO_3_ (mmol/L), and base excess in extracellular fluid (mmol/L) when compared to those of similar BW fed with the K– diets. The correlation analysis of blood chemistry response variables shown in Figures 1A–D demonstrated that the blood pH was positively correlated with blood K concentration (mmol/L) (*P* < 0.01; *R*^2^ = 0.15). The pCO_2_ (mmHg) was positively correlated with blood pH (*P* < 0.01; *R*^2^ = 0.69) and blood K (mmol/L) (*P* < 0.01, *R*^2^ = 0.13). The HCO_3_ (mmol/L) was positively correlated with pCO_2_ (mmHg) (*P* < 0.01, *R*^2^ = 0.42).

**Table 3:** Effect of limestone particle size (LPS) and dietary potassium (K) on broiler venous blood pH, sodium (Na), potassium (K), hemoglobin (Hgb), packed cell volume (PCV), glucose (Glu), ionized calcium (iCa), saturated oxygen (sO_2_), partial pressure of oxygen (pO_2_), partial pressure of carbon dioxide (pCO_2_), total carbon dioxide (TCO_2_), bicarbonate (HCO_3_), base excess in the extra cellular fluid (BEecf) at 35 days of age.

**Limestone^1^^,^^2^**	**Potassium^1^^,^^3^**	**pH**	**Na**	**K**	**Hgb**	**PCV**	**Glu**	**iCa**	**sO_**2**_**	**pO_**2**_**	**pCO_**2**_**	**TCO_**2**_**	**HCO_**3**_**	**BEecf**
			**(mmol/L)**		**(g/dl)**	**(%)**	**(mg/dl)**		**(%)**	**(mmHg)**		**(mmol/L)**		
Fine		7.41	145	5.43	7.41	21.8	229	1.37	71.9	37.8	40.4	27.0	25.8	1.31
Coarse		7.40	145	5.53	7.40	21.8	228	1.36	70.9	37.7	42.3	27.7	26.4	1.75
	K–	7.41	146^A^	5.50	7.38	21.7	235^A^	1.39^A^	71.3	37.4	41.8	27.9^a^	26.6^a^	2.13^a^
	K+	7.40	144^B^	5.47	7.44	21.9	223^B^	1.34^B^	71.6	38.1	41.0	26.8^b^	25.6^b^	0.93^b^
Fine	K–	7.42^x^	146	5.51	7.33	21.6	237	1.39	72.6	37.6	39.6^y^	27.4	26.2	2.00
Fine	K+	7.40^x^	144	5.56	7.49	22.1	222	1.35	71.2	38.0	41.2^xy^	26.6	25.4	0.62
Coarse	K–	7.40^x^	146	5.49	7.43	21.9	233	1.39	69.9	37.2	43.9^x^	28.4	27.1	2.26
Coarse	K+	7.41^x^	145	5.38	7.38	21.8	224	1.33	71.9	38.3	40.7^xy^	26.9	25.8	1.25
	SEM^4^	0.012	0.50	0.121	0.14	0.41	3.4	0.020	1.92	1.03	1.42	0.56	0.51	0.533
		(Probability > F)												
Limestone		0.54	0.52	0.41	0.96	0.98	0.82	0.72	0.59	0.94	0.20	0.19	0.18	0.38
Potassium		0.54	<0.01	0.77	0.67	0.62	<0.01	<0.01	0.87	0.48	0.58	0.03	0.04	0.02
Limestone × Potassium		0.06	0.65	0.48	0.46	0.43	0.39	0.72	0.35	0.74	0.10	0.55	0.62	0.72

x,y *Means within a column lacking a common superscript differ significantly (P < 0.10)*.

a,b *Means within a column lacking a common superscript differ significantly (P < 0.05)*.

A,B *Means within a column lacking a common superscript differ significantly (P < 0.01)*.

1 *Main effect means calculated using 64 broilers at 35 days of age*.

2 *Limestone particle size; Fine (0.2 mm) and coarse (0.9 mm)*.

3 *Potassium carbonate (starter and grower); (K–) basal levels of K; (K+) 0.20% added K*.

4 *SEM, Standard error of mean*.

### Wooden Breast and White Striping

WB and WS score results are shown in Table 4. No differences were found in WB or WS scores among broilers fed with K+ or K– diets. However, broilers fed diets with fine limestone trended toward decreased WB scores when compared to those chicks fed with coarse limestone diets (*P* < 0.10). The correlation analysis of WB scores shown in Table 5 demonstrated that BW (*P* < 0.001, *R*^2^ = 0.23) and iCa (mg/dl) (*P* < 0.01) were positively correlated with greater WB myopathy. WS was significantly correlated with _P_O_2_ (*P* < 0.02) and blood glucose (*P* ≤ 0.05).

**Table 4:** Effect of limestone particle size and dietary potassium (K) on wooden breast (WB) and white striping (WS) scores of broilers at 35 days of age.

**Limestone^1^^,^^2^**	**Potassium^1^^,^^3^**	**Wooden breast**	**White striping**
Fine		2.59^y^	2.09
Coarse		3.00^x^	2.12
	K–	2.74	2.03
	K+	2.84	2.19
Fine	K–	2.62	1.93
Fine	K+	2.56	2.25
Coarse	K–	2.85	2.13
Coarse	K+	3.12	2.12
	SEM^4^	0.228	0.144
		(Probability > F)	
Limestone		0.08	0.81
Potassium		0.66	0.29
Limestone × Potassium		0.47	0.27

x,y *Means within a column lacking a common superscript differ significantly (P < 0.10)*.

1 *Main effect means calculated using 64 broilers at 35 days of age*.

2 *Limestone particle size (starter and grower); Fine (0.2 mm) and coarse (0.9 mm)*.

3 *Potassium carbonate (starter and grower); (K–) basal levels of K; (K+) 0.20% added K*.

4 *SEM, Standard error of mean*.

**Table 5:** Broiler venous blood pH, sodium (Na), potassium (K), hemoglobin (Hgb), packed cell volume (PCV), glucose (Glu), ionized calcium (iCa), saturated oxygen (sO_2_), partial pressure of oxygen (pO_2_), partial pressure of carbon dioxide (pCO_2_), total carbon dioxide (_T_CO_2_), bicarbonate (HCO_3_), base excess in the extra cellular fluid (BEecf) at 35 days of age, in relationship to 35 days Wooden Breast (WB), and White Striping (WS) scores.

**WB^1^**	**BW**	**pH**	**Na**	**K**	**Hgb**	**PCV**	**Glu**	**iCa**	**sO_**2**_**	**pO_**2**_**	**pCO_**2**_**	**TCO_**2**_**	**HCO_**3**_**	**BEecf**
	**(g)**		**(mmol/L)**		**(g/dL)**	**(%)**	**(mg/dL)**		**(%)**	**(mmHg)**		**(mmol/L)**		
1	2,318	7.43	146	5.55	7.43	21.8	218	1.40	74.2	39.5	37.5	25.8	24.8	0.56
2	2,217	7.41	146	5.46	7.33	21.5	228	1.41	73.5	38.6	40.0	26.9	25.8	1.34
3	2,434	7.41	145	5.46	7.30	21.5	228	1.37	71.4	37.8	41.9	27.8	26.5	1.91
4	2,458	7.42	145	5.52	7.50	22.0	230	1.35	73.8	38.9	39.3	26.7	25.4	1.09
WS^2^														
1	2,301	7.42	146	5.50	7.29	21.4	231	1.39	77.7	41.5	38.8	26.7	25.4	1.07
2	2,382	7.40	145	5.48	7.42	21.8	229	1.36	71.0	37.7	41.5	27.2	26.0	1.44
3	2,387	7.43	145	5.51	7.47	21.9	219	1.39	70.9	36.9	38.8	26.6	25.5	1.16
4	–	–	–	–	–	–	–	–	–	–	–	–	–	–
SEM^3^	28	0.011	0.50	0.113	0.12	0.36	3.2	0.018	1.67	0.87	1.40	0.52	0.47	0.497
(Probability > *F*)														
WB	<0.01^*^	0.85	0.19	0.86	0.50	0.51	0.41	0.01^*^	0.95	0.97	0.90	0.81	0.92	0.94
WS	0.52	0.65	0.63	0.97	0.60	0.62	0.05^*^	0.79	0.10	0.02^*^	0.79	0.90	0.99	0.92

* *Correlation analysis differ significantly (P < 0.05)*.

1 *WB, wooden breast score (1 = none, 2 = mild, 3 = moderate, 4 = severe)*.

2 *WS, white striping score (1 = none, 2 = mild, 3 = moderate, 4 = severe)*.

3 *SEM, Standard error*.

## Discussion

The objective of the present study was to investigate the effect of dietary K supplementation and limestone particle size on live performance, blood physiology, and WS and WB myopathies. Analyzed K and DEB values (Table 1) for all diets were less than calculated, which were in agreement with Borges et al. (25). The calculated dietary K in starter and grower diets was greater than the requirement of K (0.66–0.74%) as reported by Borges et al. (26) and Oliveira et al. (27), who reported dietary K requirements for optimal BW gain to be 0.63% from 8 to 21 days of age. However, the reduction in analyzed samples compared to calculated levels may be the result of decreased K levels in feed ingredients. The formulated dietary Na (starter: 0.25%, grower: 0.23%) and Cl (starter: 0.28%, grower: 0.26%) levels were formulated to be similar; the 0.2% K_2_CO_3_ supplementation achieved the K-formulated levels of 1.03 and 0.92% in starter and grower diets, respectively. An altered DEB may have resulted in a decrease in FI and BWG, as changes in DEB and acid–base balance are linked to loss of feed consumption (13). The present findings were in agreement with Ref. (28) that reported a reduced BWG with addition of 0.15% K_2_CO_3_ and associated response to reduced water intake. However, the findings among live performance data were not in agreement with Borgatti et al. (29), who observed no growth depression by addition of K_2_CO_3_ to achieve 1.21% dietary K. The responses could be attributed to K source, i.e., K_2_CO_3_ rather than only a response to DEB or K levels *per se*. For instance, El-Deek et al. (15) reported benefits of dietary K source KCl (0.6%) on improved growth performance and broiler dressing yield.

Potassium is the major intracellular cation of the body and maintains intracellular fluid volume and acid–base balance (15). Thus, this cation is maintained within a narrow concentration in the blood (13). Ait-Boulahsen et al. (16) reported that increased plasma K was associated with higher K intake with 0.9% KCl supplementation. However, in the present study, the venous blood K was not different between the K– and K+ dietary treatments, which implied a maintenance mechanism for blood K. The K– dietary treatments exhibited elevated blood Na, which is indicative of higher ECF volume. Elevated blood glucose may have been associated with the increased FI of the birds' potentially increasing water intake. These suggested alterations in metabolic activity (30) are associated with the role of Na in glucose absorption. This somewhat explained the negative impact of K+ treatment diets on BWG. However, the decreased blood TCO_2_ suggested that there was no occurrence of hyperkalemia in K+-fed birds (13). The K– dietary treatments increased the blood iCa, which was not in agreement with Ait-Boulahsen et al. (16), who reported increased iCa with 0.6% and 0.9% KCl supplementation; however, the mechanism for increased iCa could not be completely explained without measuring the activity-specific enzymes. However, in the present study, it could only be speculated that the blood iCa changes may have been an outcome of Ca^2+^-mediated changes in muscle membrane stability (8), which may have been related to lower prevalence of WB.

The correlation analysis of blood pH and blood buffers provided a deeper insight into the acid–base status; pH, pCO_2_, and HCO_3_ (Figures 1A–D) were used in the assessment of the acid–base status. In the present study, the resting bird's venous blood chemistry values were within the range reported by Martin et al. (21). The blood pH of 7.41 and 7.40 in K– and K+-fed birds, respectively, were consistent with a homeostasis condition, and the data agreed with (31), that reported blood pH values of 7.40. There were no differences found in blood pH in the present study; however, the numerical difference between the pH could not be ignored. It somewhat explained the resting near base line acid–base status being marginally influenced by the K– and K+ dietary treatments. The blood pH was found negatively correlated with the blood K concentration. This was consistent with the findings of Ait-Boulahsen et al. (16), who reported a decrease in blood pH associated with 0.9% dietary KCl.

The K+ dietary treatments exhibited decreased blood TCO_2_, HCO_3_, and BEecf. Apparently, no respiratory compensation was exhibited as the pCO_2_ values did not differ between the dietary treatments. However, the correlation analysis between pCO_2_ and pH, (*R*^2^ = 0.69), and pCO_2_ and HCO_3_ (*R*^2^ = 0.42) explained that pCO_2_ increases accounted for compensation of elevated H+ ion concentrations and increases HCO_3_. This compensatory mechanistic relationship for maintaining acid–base homeostasis is well-documented (6), where buffer pair ratios change in unison according to the *isohydric principle* to bind the load of H+ ions; thus, the pCO_2_ increased to balance the increase in HCO_3_. The coarse limestone/K– dietary treatment tended to result in greater BWG and increased (*P* = 0.10) pCO_2_ (43.9 vs. 40.7 mmHg) when compared to K+-fed birds. The transitory reactions leading to blood gas and anion changes when not compensated may have resulted in reduced live performance in K+ diets. One notable fact was that the HCO_3_- concentration followed blood Na in K+ and K– diets, which was in agreement with Benton et al. (32). Taking growth performance and physiological responses data together, it could be implied that the blood pH (i.e., the H+ ion concentration) is a key regulatory blood biomarker that warrants further investigations to elucidate the effects of acid–base regulatory ions Na, Cl, and K, as well as their interactions with various dietary sources of these ions.

In the present study, the fine limestone-fed broilers tended to exhibit decreased WB scores (*P* = 0.08) at 35 days of age. During the grower period, the BWG in fine limestone-fed broilers trended lower (2,194 vs. 2,208 g). These results implied that a smaller BW may have been associated with decreased WB myopathies. Independent of dietary treatments, the individual BW data and WB scores were analyzed for correlations. These data indicated that greater BW was associated with higher WB score (*P* < 0.001, *R*^2^ = 0.23). This was in agreement with Kuttappan et al. (33), who reported that heavier birds have greater incidences of WB and (22) also observed that reduced BW was associated with lower prevalence of muscle myopathies. Furthermore, blood chemistry data, notwithstanding statistical significance, suggested that greater Hgb, PCV, and blood glucose in fine limestone-fed diets could be influenced by greater blood saturated O_2_ (71.9 vs. 70.9 mmHg). This reflected positive aerobic status or metabolic activity (34) and may have been associated with lower WB myopathy (22).

The interaction between blood pH (*P* = 0.06) and pCO_2_ (*P* < 0.10) demonstrated that the broilers fed with fine limestone/K– diets returned to an improved pH (7.42 vs. 7.40) compared to coarse limestone/K–. This indicated that fine limestone-fed broilers were relatively less acidotic with a lower H+ ion concentration, which in turn required lower homeostatic compensation as indicated by the blood concentration of pCO_2_ (39.63 vs. 43.87). This may have been primarily associated with the lower BWG (>59 g/bird) yielding lesser H+ ion concentrations that could have been related to decreased metabolic rate as evidenced by the reduced BWG. The correlation data demonstrated that severity of WB was correlated with lowered blood iCa levels, which have been attributed to intracellular disruption of Ca homeostasis during early onset of WB myopathies (9). Severity of WS was correlated with lowered pO_2_ and likely to be associated with hypoxia (34). This may be an effect rather than a cause mitigated by the mechanism of O_2_ extraction from tissues, which could be measured by arterial O_2_ status. The correlation of WS with decreased blood glucose levels perhaps indicated that an induced state of muscle contraction was not resolved, which may have been an early sign of onset of WS myopathy.

In conclusion, findings in the current study shows that K supplementation had a more pronounced effect on live performance and blood physiology parameters compared to limestone particle size. Results demonstrated that K supplementation as K_2_CO_3_ altered the DEB of the diets decreasing the feed intake and negatively affecting live performance. Furthermore, the physiological responses indicated that the acid–base status and blood gas parameters appeared to be in a normal homeostatic range for these broiler chickens. However, the numerical increase in the pH in K+ diets seems to be the key regulatory biomarker warranting physiological compensation. In this experiment, blood Cl was not measured. Indeed, this is an important measurement as it is involved in the maintenance of blood gas acid–base management in homeothermic animals. Thus, monitoring Cl would have made a more powerful argument in this experiment. On the other hand, feeding fine limestone assisted in lowering breast muscle myopathy, which could be associated with growth metabolism of broilers. These findings can be further supported by additional research to discern the effects at different time points. The physiological responses indicated that WB and WS myopathies were associated with inadequate respiratory gas exchange, inadequacy of Ca homeostasis, and altered blood glucose. The rate of protein accretion of breast muscle as indicated by higher growth rate is likely taking precedence over metabolic energy regulation resulting in an overall imbalance.

## Data Availability Statement

The raw data supporting the conclusions of this article will be made available by the authors, without undue reservation.

## Ethics Statement

The animal study was reviewed and approved by Institutional Animal Care and Use Committee North Carolina State University.

## Author Contributions

DJ wrote the manuscript. All authors listed have made a substantial contribution to the design, conducting, preparing, and reviewing of the manuscript.

## Conflict of Interest

The authors declare that the research was conducted in the absence of any commercial or financial relationships that could be construed as a potential conflict of interest.
